# Unexpected Inheritance Pattern of *Erianthus arundinaceus* Chromosomes in the Intergeneric Progeny between *Saccharum* spp. and *Erianthus arundinaceus*

**DOI:** 10.1371/journal.pone.0110390

**Published:** 2014-10-13

**Authors:** Jiayun Wu, Yongji Huang, Yanquan Lin, Cheng Fu, Shaomou Liu, Zuhu Deng, Qiwei Li, Zhongxing Huang, Rukai Chen, Muqing Zhang

**Affiliations:** 1 Key Lab of Sugarcane Biology and Genetic Breeding, Ministry of Agriculture, Fujian Agriculture and Forestry University, Fuzhou, China; 2 Guangzhou Research Institute for Sugarcane Industry, Guangzhou, China; 3 State Key lab for Conservation and Utilization of Subtropical Agro-bioresources, Guangxi University, Nanning, China; United States Department of Agriculture, United States of America

## Abstract

*Erianthus arundinaceus* is a valuable source of agronomic traits for sugarcane improvement such as ratoonability, biomass, vigor, tolerance to drought and water logging, as well as resistance to pests and disease. To investigate the introgression of the *E*. *arundinaceus* genome into sugarcane, five intergeneric F_1_ hybrids between *S. officinarum* and *E*. *arundinaceus* and 13 of their BC_1_ progeny were studied using the genomic in situ hybridization (GISH) technique. In doing so, we assessed the chromosome composition and chromosome transmission in these plants. All F_1_ hybrids were aneuploidy, containing either 28 or 29 *E*. *arundinaceus* chromosomes. The number of *E*. *arundinaceus* chromosomes in nine of the BC_1_ progeny was less than or equal to 29. Unexpectedly, the number of *E*. *arundinaceus* chromosomes in the other four BC_1_ progeny was above 29, which was more than in their F_1_ female parents. This is the first cytogenetic evidence for an unexpected inheritance pattern of *E*. *arundinaceus* chromosomes in sugarcane. We pointed to several mechanisms that may be involved in generating more than 2n gametes in the BC_1_ progeny. Furthermore, the implication of these results for sugarcane breeding programs was discussed.

## Introduction

Sugarcane (*Saccharum* spp.) plays a pivotal role in world agriculture as a primary sugar-producing crop and has significant potential as a renewable bioenergy crop [1]. The genus *Saccharum* is comprised of six species: The two wild species are *S*. *spontaneum* and *S*. *robustum*, and the four cultivated species are *S*. *officinarum*, *S*. *barberi*, *S*. *sinense* and *S*. *edule*. *S*. *officinarum* (2n = 80) is known as the noble cane due to its high sugar content and thick and juicy culms. The wild species *S*. *spontaneum* (2n = 40–128; chromosome number varies) has very low sugar content but exhibits high vigor, profuse tillers and strong ratooning ability, as well as resistance to diseases and pests. Modern sugarcane cultivars are highly complex polyploid aneuploids and typically have 100–130 chromosomes derived from a combination of these two species [2].

During the early 20^th^ century, interspecific hybridization was used to introgress desirable traits from wild species into sugarcane cultivars, and this practice led to substantial improvements in sugarcane agriculture [3]. In particular, interspecific hybridization between *S*. *officinarum* as the female parent and *S*. *spontaneum* as the male parent, followed by successive backcrosses of the hybrids to different clones of *S*. *officinarum* as the recurrent parent, significantly increased cane yields and resistance to biotic and abiotic stresses. In this hybridization strategy, F_1_ hybrids and plants in the first backcross generation (BC_1_) receive 2n gametes from female parent and n gametes from male parent, and plants in the second backcross generation (BC_2_) receives n gametes from both the female and male parents [4]. The purpose of the process termed nobilization, which refers to the crossing and backcrossing of intergenic hybrids to noble cane, was to retain high-sugar producing clones and to eliminate the negative effects of wild germplasm [5]. Over the past decades, much insight has been gained into the mechanisms underlying 2n gamete formation through the use of molecular genetic and cytological techniques. Using simple sequence repeat (SSR), amplified fragment length polymorphism (AFLP) and diversity arrays technology (DarT), Hermann et al. [6] provided molecular marker data that suggested the mechanism was second division restitution (SDR) or megaspore tetrad cell fusion (MTCF). Bielig et al. [7] provided cytological evidence that 2n male gamete formation was probably attributable to SDR. Nevertheless, the mechanisms underlying 2n gamete formation in sugarcane are still not fully understood.

Indeed, the occurrence of 2n gametes is not rare in the plant kingdom and it has been reported in many genera, including *Brassica*, *Paspalum*, *Brachiaria*, *Citrus*, *Fragaria*, *Malus*, *Manihot*, *Medicago*, *Solanum*, and *Trifolium* [8]–[11]. Several explanations for 2n gamete formation have been proposed, including pre-meiotic and post-meiotic genome doubling, and meiotic restitution. Among these possibilities, the majority of reports have identified a restitution of the meiotic cell cycle in several species [12]–[14], suggesting that it is the predominant mechanism of 2n gamete formation in plants. However, a small number of reports have documented pre-meiotic and post-meiotic genome duplications, indicating that these mechanisms of 2n gamete formation are quite rare. Three cytological processes can lead to 2n gamete formation during abnormal meiosis: first division restitution (FDR), second division restitution (SDR) and indeterminate meiotic restitution (IMR). In FDR, homologous chromosomes remain together when the nucleus fails to divide after telophase I, and after a normal second division, sister chromatids derived from each chromosome move to opposite poles. In SDR, normal separation of the homologous chromosomes at first division is followed by the absence of the second meiotic division and sister chromatids fail to migrate to opposite poles at second division. IMR has been best described in lily meiocytes, and simultaneously shows characteristics similar to both SDR and FDR within a single meiocyte [15]–[18].

Modern sugarcane cultivars have limited genetic diversity, due to the small number of progenitors used in the initial interspecific hybridizations during the process of nobilization [19]–[21]. This genetic bottleneck has impeded further sugarcane improvement for certain traits such as tolerance to biotic and abiotic stresses. Therefore, it is urgent to broaden the genetic base of sugarcane by introgressing favorable genes from closely related *Erianthus*, *Miscanthus*, *Narenga* and *Sclerostachya* genera [22].

*Erianthus* is one of the most closely related genera to *Saccharum* and has attracted the interest of sugarcane breeders worldwide. *Erianthus arundinaceus* (*E*. *arundinaceus*, 2n = 20, 40, 60) is one of eight species in the genus *Erianthus* [23], and it possesses valuable agronomic traits for sugarcane improvement such as high biomass, vigor, ratoonability, tolerance to drought and water logging, and resistance to pests and disease [23]–[28]. Favorable alleles can be introduced into modern sugarcane cultivars for yield and stability improvement, although the hybrid progeny is often sterile [26]. However, significant progress has been made to produce genuine F_1_ hybrids and to backcross the progeny successfully. Molecular markers and genomic in situ hybridization (GISH) techniques have been used to identify true intergeneric hybrids between *Saccharum* spp. and *Erianthus* spp. [23], [24], [26], [29]. According to histological staining character of root tips, Fukuhara et al. [30] concluded that F_1_ hybrids were successfully obtained from the intergeneric hybridization between *Saccharum* spp. hybrid and *E*. *arundinaceus*.

Using GISH, N. Piperidis et al. [29] reported that chromosome transmission was n+n in both F_1_ (*S*. *officinarum* × *E*. *arundinaceus*) and BC_2_ (BC_1_ × sugarcane cultivar) generations, but was 2n+n in the BC_1_ (F_1_ × sugarcane cultivar) cross. In similar crosses six F_1_ hybrids had fewer than 70 chromosomes and one had more than 70, indicating that all F_1_ crosses were aneuploidy [26]. In this report, we studied chromosome transmission in (*E. arundinaceus × S. officinarum*) hybrids by using GISH to determine the chromosome composition of two generations including five intergeneric F_1_ hybrids and 13 BC_1_ progeny.

## Materials and Methods

### Plant materials

The plant materials used in this study consisted of 18 clones derived from two generations of intergeneric hybrids (Table 1). The male parent of the F_1_ generation was either *E. arundinaceus* HN 92-77 (2n = 60) or HN 92–105 (2n = 60) from Hainan, China. *S. officinarum* Badila (2n = 80) was used as the female parent for the F_1_ generation. Female parents of the BC_1_ generation were YCE 95-41, YCE 96-40 and YCE 96-66, which were derived from crosses between Badila and HN 92-77 or HN 92–105. The male parent of the BC_1_ generation was CP 84–1198 (2n = 120), which is a commercial cultivar containing germplasm from *S. officinarum*, *S. spontaneum*, *S. barberi* and *S. robustum* without contribution from *E. arundinaceus.* F_1_ and BC_1_ plants were generated at the Hainan Sugarcane Breeding Station of Guangzhou Sugarcane Industry Research Institute. All clones were planted in the greenhouse at Fujian Agriculture and Forestry University.

**Table 1:** The intergeneric F_1_ hybrids and their BC_1_ progeny between *Saccharum* spp. and *E*. *arundinaceus.*

Generation	Clones	Female parent	Male parent
F_1_	YCE 96-66	Badila	HN 92-105
F_1_	YCE 96-40	Badila	HN 92-77
F_1_	YCE 96-43	Badila	HN 92-77
F_1_	YCE 96-45	Badila	HN 92-77
F_1_	YCE 95-41	Badila	HN 92-77
BC_1_	YCE 01-33	YCE 95-41	CP 84-1198
BC_1_	YCE 01-46	YCE 95-41	CP 84-1198
BC_1_	YCE 01-48	YCE 95-41	CP 84-1198
BC_1_	YCE 01-36	YCE 96-40	CP 84-1198
BC_1_	YCE 01-92	YCE 96-40	CP 84-1198
BC_1_	YCE 01-99	YCE 96-40	CP 84-1198
BC_1_	YCE 01-102	YCE 96-40	CP 84-1198
BC_1_	YCE 01-105	YCE 96-40	CP 84-1198
BC_1_	YCE 01-116	YCE 96-40	CP 84-1198
BC_1_	YCE 01-134	YCE 96-40	CP 84-1198
BC_1_	YCE 01-63	YCE 96-66	CP 84-1198
BC_1_	YCE 01-61	YCE 96-66	CP 84-1198
BC_1_	YCE 01-69	YCE 96-66	CP 84-1198

### Genomic in situ hybridization procedure

Chromosome preparation, chromosome spreading and GISH experiments were performed as described in D’hont et al. [24]. Genomic DNA from *E. arundinaceus* HN 92-77 and HN 92–105 was labeled with digoxigenin-11-dUTP (Roche) and genomic DNA from Badila and CP 84–1198 was labeled with biotin-16-dUTP (Roche) using the Nick Translation Kit (Roche). To detect signal from biotin-labeled probes, Avidin D, Rhodamine 600 (XRITC) and biotinylated anti-avidin antibody (Vector Laboratories, Burlingame, CA) were used. To detect signal from digoxigenin-labeled probes, sheep-anti-digoxin-FITC (Roche, Lewes, UK) and rabbit-anti-sheep-FITC (Roche, Lewes, UK) were used. Chromosomes were then counter stained using DAPI in Vectashield anti-fade solution Vectashield (Vector Laboratories, Burlingame, CA). The hybridization signals were observed on an AxioScope A1 Imager fluorescent microscope (Carl Zeiss, Gottingen, Germany). Images were captured digitally with an AxioCam MRc5 and AxioVision v.4.7 imaging software (Carl Zeiss, Gottingen, Germany).

## Results and Discussion

### Aneuploidy in F_1_ hybrids

**Figure 1:**
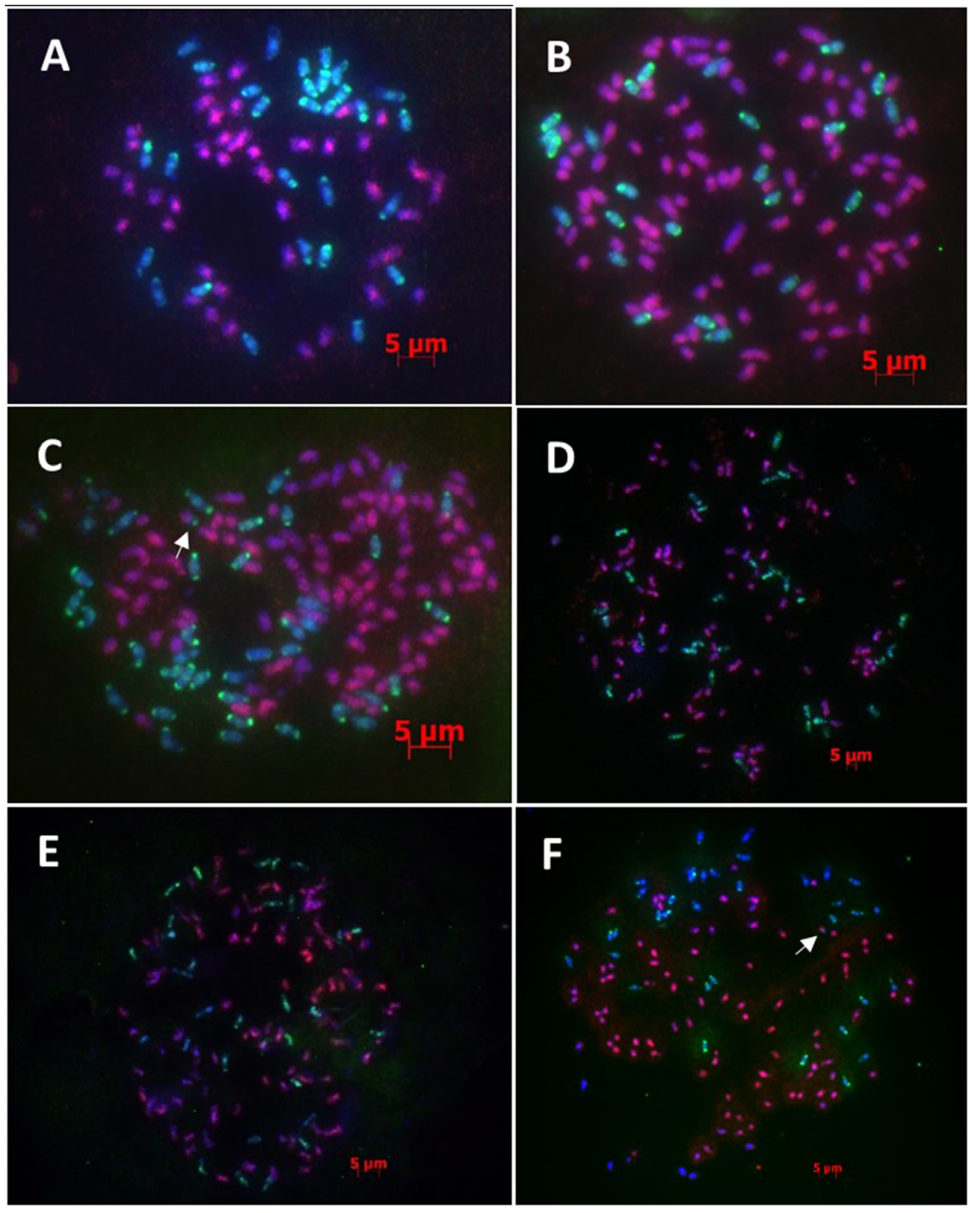
GISH analysis of the F_1_ hybrids and BC_1_ progeny. *Saccharum* spp. chromosomes were visualized in red and *E*. *arundinaceus* chromosomes in green. (A) YCE 96-66 (F_1_): 29 chromosomes from *E*. *arundinaceus* and 40 chromosomes from *Saccharum* spp.; (B) YCE 01–102 (BC_1_): 22 chromosomes from *E*. *arundinaceus* and 96 chromosomes from *Saccharum* spp.; (C) YCE 01–36 (BC_1_): 36 chromosomes from *E*. *arundinaceus*, 96 chromosomes from *Saccharum* spp. and one terminally translocated chromosome; (D) YCE 01–61 (BC_1_): 31 chromosomes from *E*. *arundinaceus* and 85 chromosomes from *Saccharum* spp.; (E) YCE 01–69 (BC_1_): 31 chromosomes from *E*. *arundinaceus* and 88 chromosomes from *Saccharum* spp.; (F) YCE 01–92 (BC_1_): 35 chromosomes from *E*. *arundinaceus*, 95 chromosomes from *Saccharum* spp. and one terminally translocated chromosome. The arrowhead in Figure 1C and Figure 1F shows the translocated chromosome. Scale bars: 5 µm.

Five F_1_ hybrids analyzed by GISH were characterized by the presence of 68–69 chromosomes, consisting of 40 *Saccharum*-derived chromosomes and 28–29 *E*. *arundinaceus*-derived chromosomes (Figure 1A, Figure S1–S4 in File S1). Therefore, the five F_1_ hybrids were the products of n+n chromosome transmission (Table 2), and all hybrids were also aneuploid. These results are consistent with G. Piperidis’s studies [26]. Notably, the high rate of aneuploidy in F_1_ hybrids contributes to the production of unbalanced gametes, which might be associated with the high degree of sterility in F_1_ hybrids [31], [32]. Chromosomes inherited from divergent parents are often unable to pair with each other in meiosis [33]–[36], producing very few or no viable pollen grains [26], [37]. In order to obtain backcross generations, an adjustment in sugarcane breeding was implemented, involving F_1_ hybrids as female parents and CP 84-1198 as the male parent, although this does not conform perfectly to the fundamental principles of backcross breeding. Even though F_1_ hybrids were used as female parents, their fertility was still very low. Among the five F_1_ hybrids, only YCE 96-40, YCE 96-66 and YCE 95-41 as female parents generated several BC_1_ progeny by backcrossing with CP 84–1198. Attempts to backcross YCE 96-43 and YCE 96-45 to CP 84-1198 were not successful.

**Table 2:** Chromosome composition of F_1_ hybrids and BC_1_ progeny.

Generation	Clones	Chromosome Composition							No. of cells observed
		Modal number Range		Modal number Range		Modal number Range		Recombinants	
		2n cell		*Saccharum* spp.		*E. arundinaceus*			
F_1_	YCE 95-41	68	68–70	40	40	28	28–30	0	4
F_1_	YCE 96-40	69	68–70	40	40	29	28–30	0	21
F_1_	YCE 96-43	69	68–70	40	40	29	28–30	0	4
F_1_	YCE 96-45	69	67–70	40	40	29	27–30	0	12
F_1_	YCE 96-66	69	67–70	40	40	29	27–30	0	22
BC_1_	YCE 01-33	120	118–121	93	91–93	27	26–28	0	5
BC_1_	YCE 01–46	125	122–127	96	95–97	29	28–29	0	6
BC_1_	YCE 01-48	120	117–121	93	93–94	27	26–29	0	15
BC_1_	YCE 01-63	125	123–126	97	95–98	28	26–28	0	8
BC_1_	YCE 01-99	118	117–120	95	95–97	23	21–23	0	4
BC_1_	YCE 01-102	118	115–119	96	94–97	22	19–24	0	23
BC_1_	YCE 01-105	117	115–119	94	93–96	23	22–23	0	12
BC_1_	YCE 01-116	122	119–124	94	92–95	28	26–29	0	10
BC_1_	YCE 01-134	121	120–122	93	93–95	28	26–29	0	8
BC_1_	YCE 01-36	134	130–136	96	95–97	36	35–36	1	10
BC_1_	YCE 01-92	130	129–132	95	94–96	35	34–36	1	8
BC_1_	YCE 01-61	116	114–118	85	84–85	31	30–31	0	3
BC_1_	YCE 01-69	119	115–120	88	87–90	31	29–32	0	8

**Note:** The modal number of chromosomes is presented for the sugarcane clones analysed, since small variation of chromosome counts can occur due to the loss or the overlapping of a few chromosomes from the preparation.

### Unexpected inheritance pattern in BC_1_ progeny

GISH analysis of nine BC_1_ progeny revealed plants with a total chromosome complement ranging from 117 to 125 (Table 2), of which 93 to 97 chromosomes were derived from *Saccharum* and 22 to 29 chromosomes were derived from *E*. *arundinaceus* (Figure 1B, Figure S5–S12 in File S1). These results indicated that the nine BC_1_ progeny were products of 2n+n transmission. G. Piperidis et al. [26] and N. Piperidis et al. [29] reported similar results. GISH analysis of another four BC_1_ progeny revealed plants with a total chromosome complement ranging from 116 to 132, and evidence of an unusual mode of chromosome transmission (Table 2, Figure 1C–1F). In YCE 01–36, YCE 01–61, YCE 01–69 and YCE 01–92, 85 to 96 chromosomes were derived from *Saccharum* and 36, 31, 31 and 35 chromosomes were derived from *E*. *arundinaceus*, respectively. These results indicated that, in these four BC_1_ progeny, more than 29 *E*. *arundinaceus*-derived chromosomes (Table 2, Figure 1) were transmitted, which is a greater number than was detected in the F_1_ generation. To our knowledge, ours is the first report to document that the *E*. *arundinaceus*-derived chromosome number was above 29 in BC_1_ progeny. Within the plant kingdom, this unusual phenomenon has rarely been reported. It is especially noteworthy that four BC_1_ progeny out of 13 exhibited greater than 2n female-inherited chromosomes, suggesting that this newly discovered phenomenon can occur at relatively high frequency in sugarcane (above 30%). More importantly, the occurrence of more than 2n female gametes was detected in two diverse parental combinations rather than an individual case, suggesting that this phenomenon is not restricted to an individual plant. The results also suggest that the four BC_1_ progeny were the product of a new pattern of chromosome transmission. Interestingly, YCE 01–36 (Figure 1C) and YCE 01–92 (Figure 1F) were found to both have a terminally translocated chromosomes.

### Possible mechanisms

**Figure 2:**
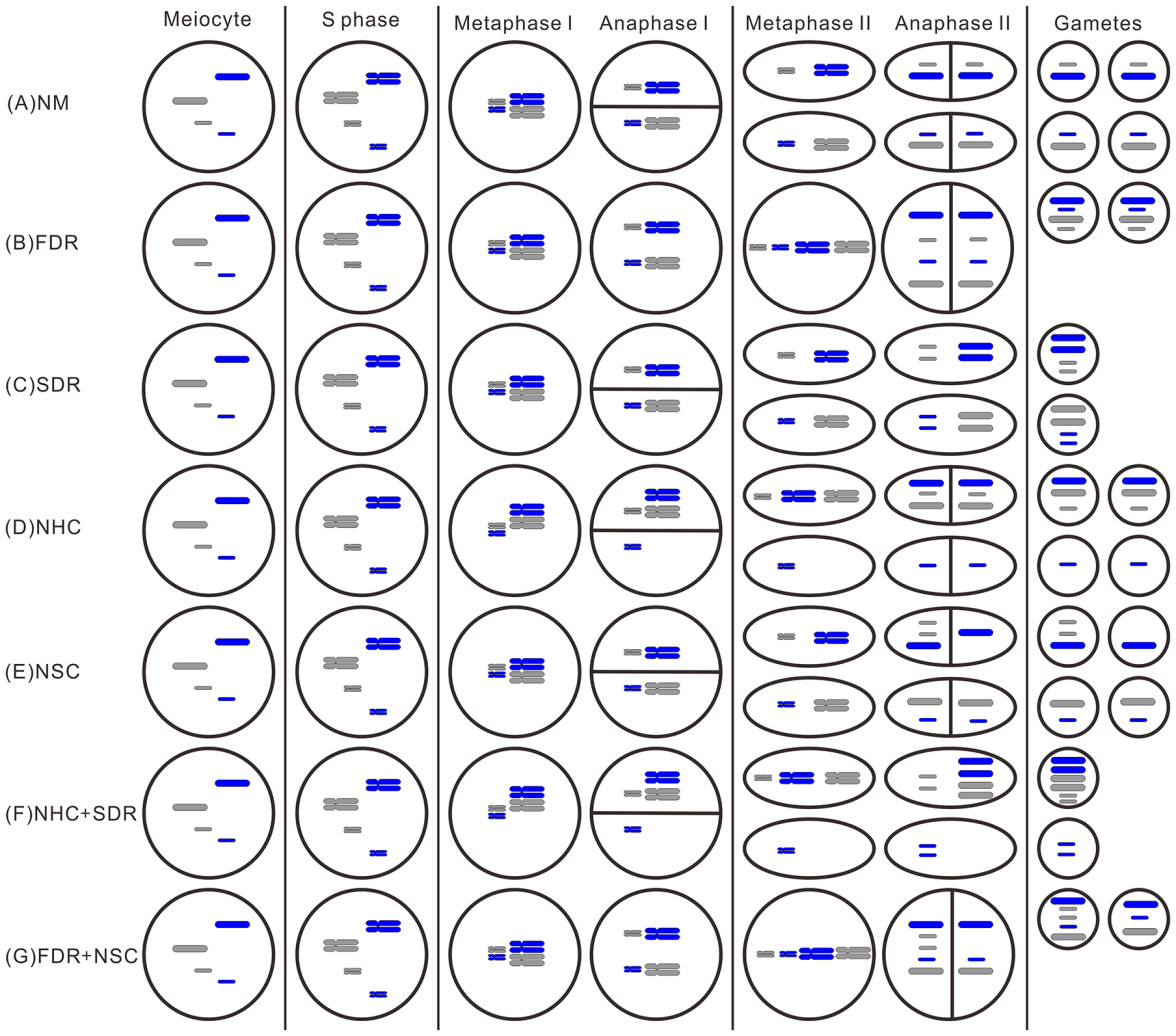
Schematic of seven meiosis scenarios with two pairs of homologous chromosomes. (A) NM: normal meiosis; (B) FDR: first meiotic division restitution; (C) SDR: second meiotic division restitution; (D) NHC: nondisjunction of homologous chromosomes in first meiotic division; (E) NSC: nondisjunction of sister chromatids in second meiotic division; (F) NHC+SDR: nondisjunction of homologous chromosomes in first meiotic division and second division restitution; (G) FDR+NSC: first division restitution and nondisjunction of a chromosome with sister chromatids in the second meiotic division. For simplicity, recombination events are not illustrated in these meiosis schematics.

The unexpected inheritance pattern that we observed in BC_1_ progeny is not in accordance with prevailing theories of chromosome transmission in hybrids. If meiosis occurs normally, four gametes are generated with different numbers of *E*. *arundinaceus*-derived chromosomes, due to the 29 *E*. *arundinaceus* chromosomes in F_1_ hybrids. As a result, two gametes are produced containing 14 *E*. *arundinaceus* chromosomes and the other two gametes contain 15 *E*. *arundinaceus* chromosomes. (Figure 2A). In FDR, two gametes are produced with 29 *E*. *arundinaceus* chromosomes (Figure 2B). In SDR, one gamete is produced with 28 *E*. *arundinaceus* chromosomes and a second contains 30 *E*. *arundinaceus* chromosomes (Figure 2C). SDR has been extensively reported in sugarcane [7], [38], [39]. In addition, Narayanaswami [40] discovered that 2n gametes originated from the fusion of the two innermost cells of the megaspore tetrad (megaspore tetrad cell fusion, MTCF). Post-meiotic restitution (PMR), in which chromosome doubling occurs after the second meiotic division, was observed by Bremer [38].

The normal separation of chromosomes in the first meiotic division or sister chromatids in the second meiotic division is called disjunction. Nondisjunction can occur in the first meiotic division (nondisjunction of homologous chromosomes; NHC) or second meiotic division (nondisjunction of sister chromatids; NSC). These distinct processes of nondisjunction create gametes with different numbers of chromosomes (Figure 2D, Figure 2E). In this study, we propose two possible mechanisms responsible for the formation of gametes with chromosome number greater than 2n. The first possibility (Model I) involves both NHC and SDR, which would generate two gametes with different even numbers of *E*. *arundinaceus* chromosomes after meiosis (Figure 2F; NHC + SDR). The second possibility (Model II) involves both FDR and NSC, which would generate two gametes with different odd or even numbers of *E*. *arundinaceus* chromosomes after meiosis (Figure 2G; FDR + NSC).

**Figure 3:**
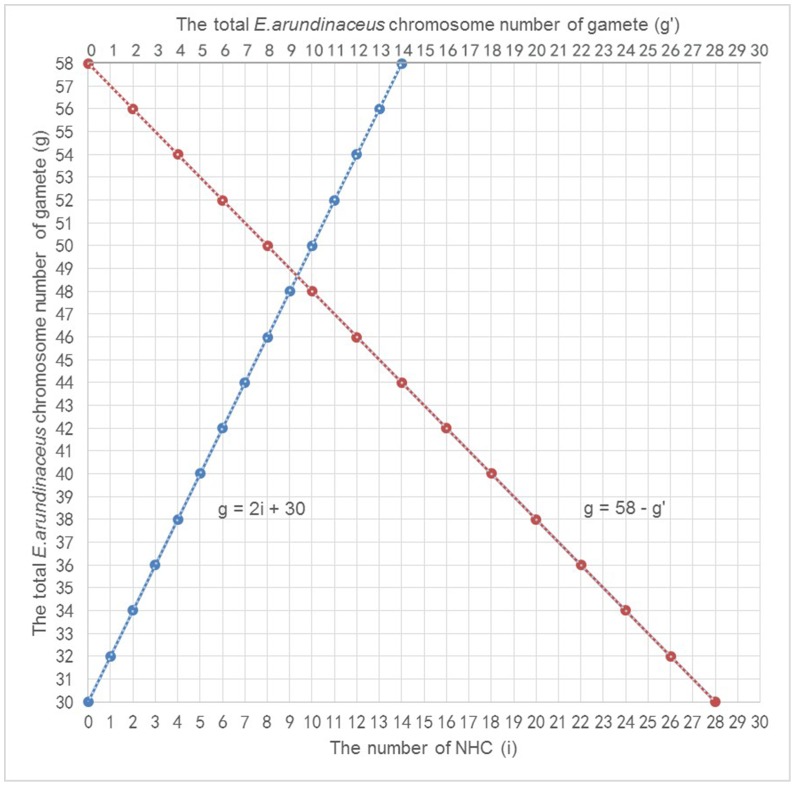
The relationship between NHC and the total *E*. *arundinaceus* chromosome number of gametes (blue curve), and the interrelationship of two gametes (red curve). Note: g and g’ are the total number of *E*. *arundinaceus* chromosomes in each gamete after meiosis, i is the number of NHC.

**Figure 4:**
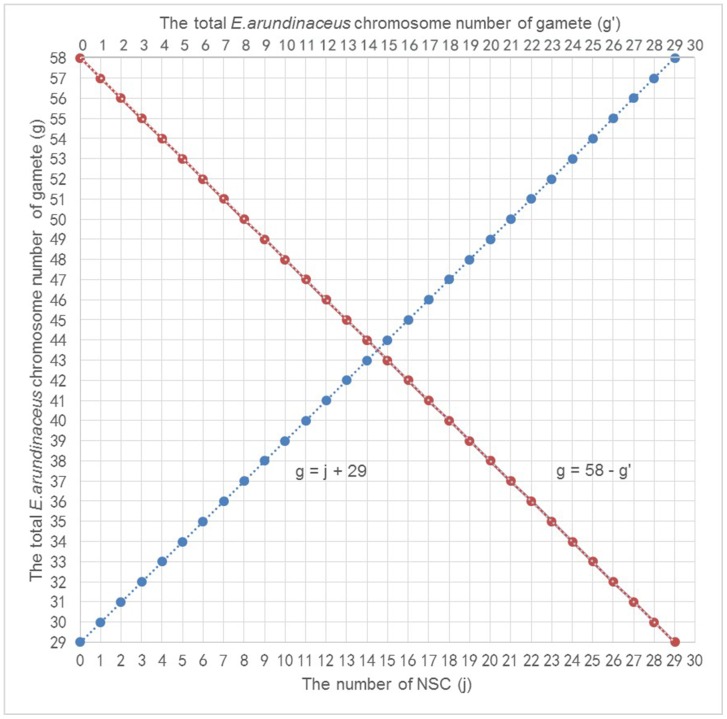
The relationship between NSC and the total number of *E*. *arundinaceus* chromosomes in each gamete (blue curve), and the interrelationship of two gametes (red curve). Note: g and g’ are the total number of *E*. *arundinaceus* chromosomes in each gamete after meiosis, j is the number of NSC.

According to the results obtained from plants in the F_1_ generation, their meiocytes contained 29 *E*. *arundinaceus* chromosomes. During S phase of pre-meiotic interphase, all chromosomes are duplicated and each chromosome is comprised of two sister chromatids. Consequently, after meiosis, the total number of *E*. *arundinaceus* chromosomes in the F_1_ gametes should be 58. According to Model I, if there are *i* pair(s) of NHC in the first meiotic division, this yields a difference of 2(2*i* +1) *E*. *arundinaceus* chromosomes between the two gametes. According to Model II, if there are *j* chromosomes with NSC in the second meiotic division, this yields a difference of 2*j E*. *arundinaceus* chromosomes between the two gametes. Thus, the following two simultaneous linear equations are obtained:
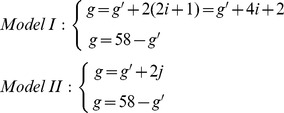



In these equations, *g* and *g’* are the total number of *E*. *arundinaceus* chromosomes in each gamete after meiosis, and *i* and *j* are the number of NHC and NSC, respectively. From our experimental observations of 29 *E*. *arundinaceus* chromosomes in a meiocyte and 58 *E*. *arundinaceus* chromosomes in two gametes, we required that 0 ≤ *g* ≤ 58; 0 ≤ *g’* ≤ 58; 0 ≤ *i* ≤ 14; and 0 ≤ *j* ≤ 29. The four variables *g*, *g’*, *i* and *j* were integral. After solving these simultaneous linear equations, we obtained two formulas:
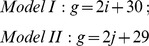



Based on these formulas, graphs of these linear equations are shown in Figure 3 and Figure 4.

In this study, eight different *E*. *arundinaceus* chromosome numbers were observed (22, 23, 27, 28, 29, 31, 35 and 36) in BC_1_ progeny. Due to the fact that we detected odd numbers of *E*. *arundinaceus* chromosomes in BC_1_ progeny, we speculate that Model II may be a more likely mechanism than Model I, but Model I cannot be ruled out as a mechanism occurring in plants that inherited even numbers of *E*. *arundinaceus* chromosomes. It is possible that both models are valid, suggesting that both mechanisms can occur.

Modern sugarcane cultivars are characterized by a high degree of inbreeding depression, so any increase in the heterozygosity of gametes may be beneficial to breeding efforts [6]. Depending on the specific mode of chromosome segregation, gametes can exhibit different degree of heterozygosity. During normal meiosis, crossing-over occurs between two non-sister chromatids. In FDR, 2n gametes always possess two non-sister chromatids and consequently maintain equivalent levels of parental heterozygosity and epistatic interactions. In SDR, sister chromatids do not separate and these gametes exhibit high levels of homozygosity. As a result, most parental heterozygosity and epistatic interactions are lost [41], [42]. The highest degree of heterogeneity is found in gametes originated through IMR, since these gametes result from a mixture of FDR and SDR [15]. In addition, when chromatids migrate to the same pole as in NHC and NSC, chromosomes are doubled. In NHC+SDR or FDR+NSC, some *E. arundinaceus* chromosomes would be doubled twice. This process likely creates a larger number of new multilocus allelic combinations and provides the opportunity to select the resulting germplasm for new, desirable traits.

### Future directions

In order to understand the underlying mechanisms involved in generating the number of *E. arundinaceus* chromosomes in BC_1_ progeny, detailed cytological observations of female gametes and chromosomal dynamics in the embryo sac of F_1_ hybrids are needed. Although difficult to access, a more thorough understanding of the megagametophyte may result in possible applications for improving sugarcane through 2n gamete transmission.

## Supporting Information

File S1 **Supporting Information Figures.** Figure S1. YCE 95-41(F_1_): 28 chromosomes from *E*. *arundinaceus* and 40 chromosomes from *Saccharum* spp. Figure S2. YCE 96-40(F_1_): 29 chromosomes from *E*. *arundinaceus* and 40 chromosomes from *Saccharum* spp. Figure S3. YCE 96-43(F_1_): 29 chromosomes from *E*. *arundinaceus* and 40 chromosomes from *Saccharum* spp. Figure S4. YCE 96-45(F_1_): 29 chromosomes from *E*. *arundinaceus* and 40 chromosomes from *Saccharum* spp. Figure S5. YCE 01–33 (BC_1_): 27 chromosomes from *E*. *arundinaceus* and 93 chromosomes from *Saccharum* spp. Figure S6. YCE 01–46 (BC_1_): 29 chromosomes from *E*. *arundinaceus* and 96 chromosomes from *Saccharum* spp. Figure S7. YCE 01–48 (BC_1_): 27 chromosomes from *E*. *arundinaceus* and 93 chromosomes from *Saccharum* spp. Figure S8. YCE 01–63 (BC_1_): 28 chromosomes from *E*. *arundinaceus* and 97 chromosomes from *Saccharum* spp. Figure S9. YCE 01–99 (BC_1_): 23 chromosomes from *E*. *arundinaceus* and 95 chromosomes from *Saccharum* spp. Figure S10. YCE 01–105 (BC_1_): 23 chromosomes from *E*. *arundinaceus* and 94 chromosomes from *Saccharum* spp. Figure S11. YCE 01–116 (BC_1_): 28 chromosomes from *E*. *arundinaceus* and 94 chromosomes from *Saccharum* spp. Figure S12. YCE 01–134 (BC_1_): 28 chromosomes from *E*. *arundinaceus* and 93 chromosomes from *Saccharum* spp.
(DOC)
