# Supplementary material for: Unexpected Inheritance Pattern of Erianthus arundinaceus Chromosomes in the Intergeneric Progeny between Saccharum spp. and Erianthus arundinaceus
Source: PLoS One. 2014 Oct 13;9(10):e110390. doi: 10.1371/journal.pone.0110390 (PMC4195721; doi:10.1371/journal.pone.0110390)
Supplement: File S1 — Supporting Information Figures. Figure S1. YCE 95-41(F1): 28 chromosomes from E. arundinaceus and 40 chromosomes from Saccharum spp. Figure S2. YCE 96-40(F1): 29 chromosomes from E. arundinaceus and 40 chromosomes from Saccharum spp. Figure S3. YCE 96-43(F1): 29 chromosomes from E. arundinaceus and 40 chromosomes from Saccharum spp. Figure S4. YCE 96-45(F1): 29 chromosomes from E. arundinaceus and 40 chromosomes from Saccharum spp. Figure S5. YCE 01–33 (BC1): 27 chromosomes from E. arundinaceus and 93 chromosomes from Saccharum spp. Figure S6. YCE 01–46 (BC1): 29 chromosomes from E. arundinaceus and 96 chromosomes from Saccharum spp. Figure S7. YCE 01–48 (BC1): 27 chromosomes from E. arundinaceus and 93 chromosomes from Saccharum spp. Figure S8. YCE 01–63 (BC1): 28 chromosomes from E. arundinaceus and 97 chromosomes from Saccharum spp. Figure S9. YCE 01–99 (BC1): 23 chromosomes from E. arundinaceus and 95 chromosomes from Saccharum spp. Figure S10. YCE 01–105 (BC1): 23 chromosomes from E. arundinaceus and 94 chromosomes from Saccharum spp. Figure S11. YCE 01–116 (BC1): 28 chromosomes from E. arundinaceus and 94 chromosomes from Saccharum spp. Figure S12. YCE 01–134 (BC1): 28 chromosomes from E. arundinaceus and 93 chromosomes from Saccharum spp. (DOC) [file pone.0110390.s001.doc]

**File S1**

**Figure S1**

**
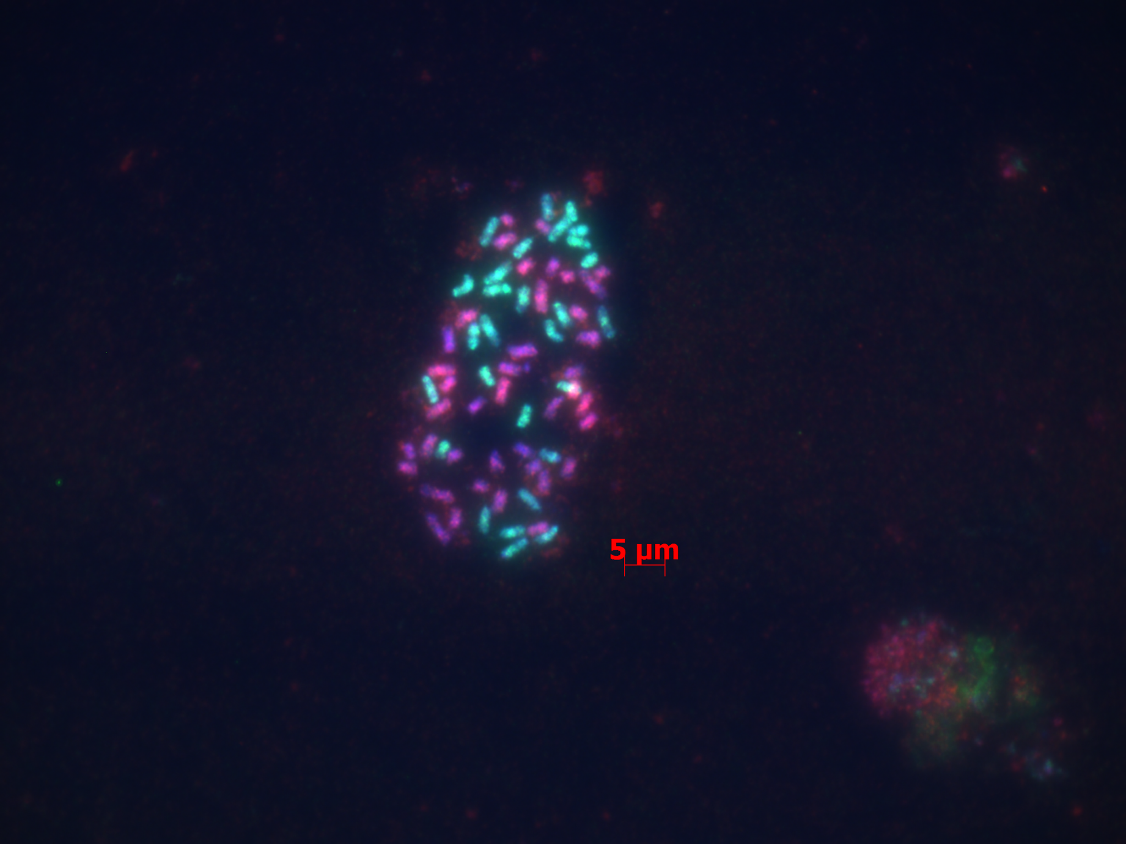
**

**YCE 95-41(F_1_)**: 28 chromosomes from *E*. *arundinaceus* and 40 chromosomes from *Saccharum* spp.

**Figure S2**


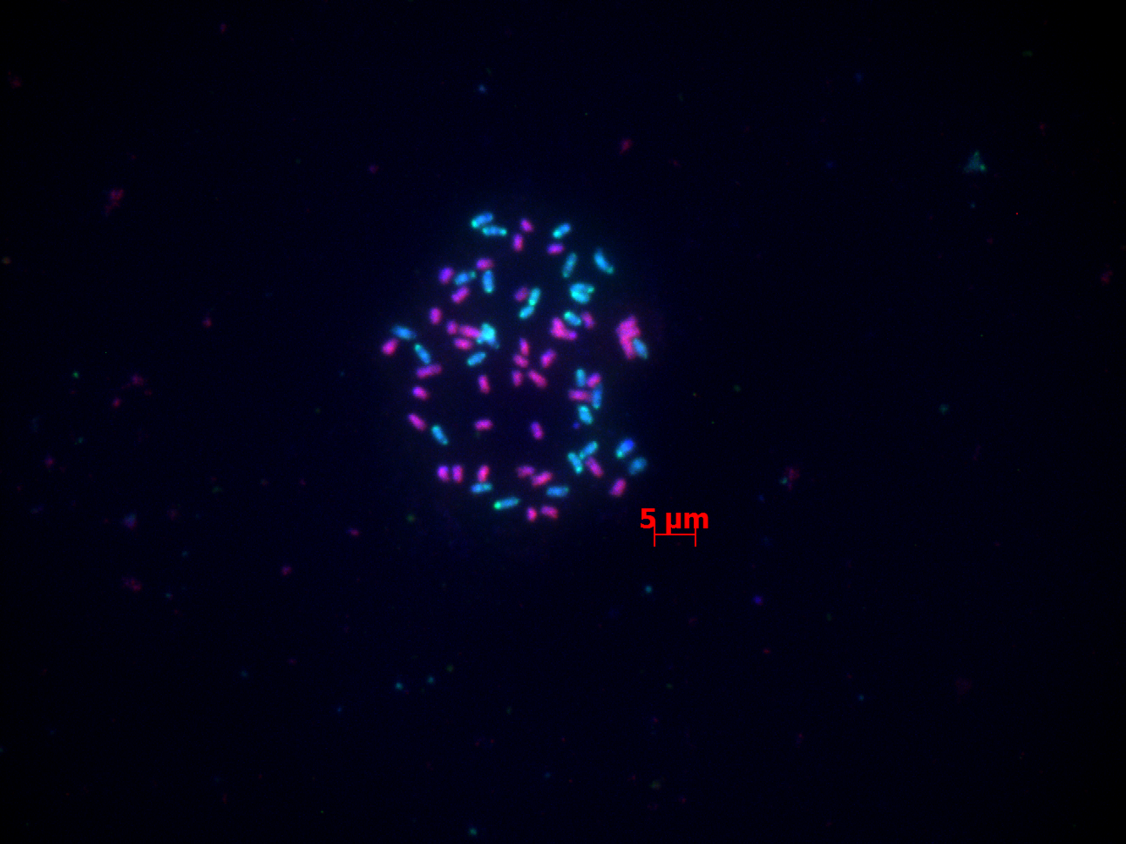


**YCE 96-40(F_1_)**: 29 chromosomes from *E*. *arundinaceus* and 40 chromosomes from *Saccharum* spp.

**Figure S3**


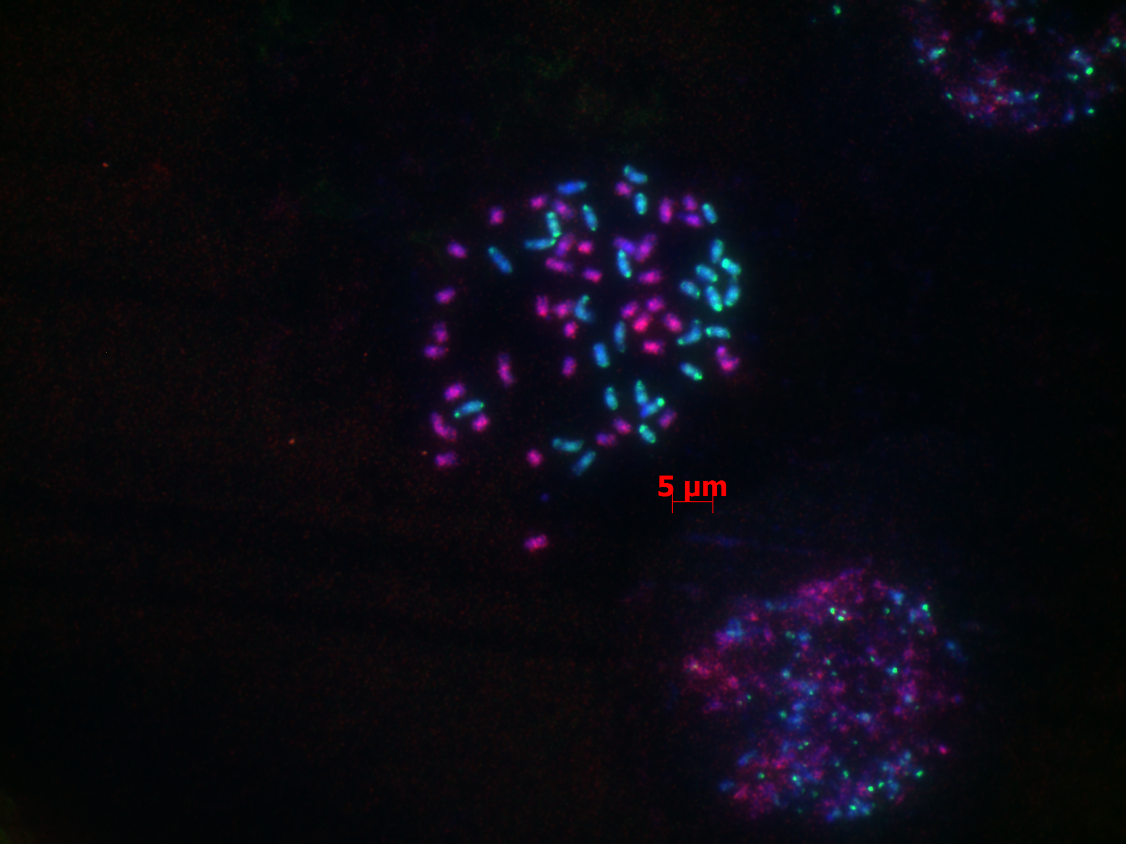


**YCE 96-43(F_1_)**: 29 chromosomes from *E*. *arundinaceus* and 40 chromosomes from *Saccharum* spp.

**Figure S4**


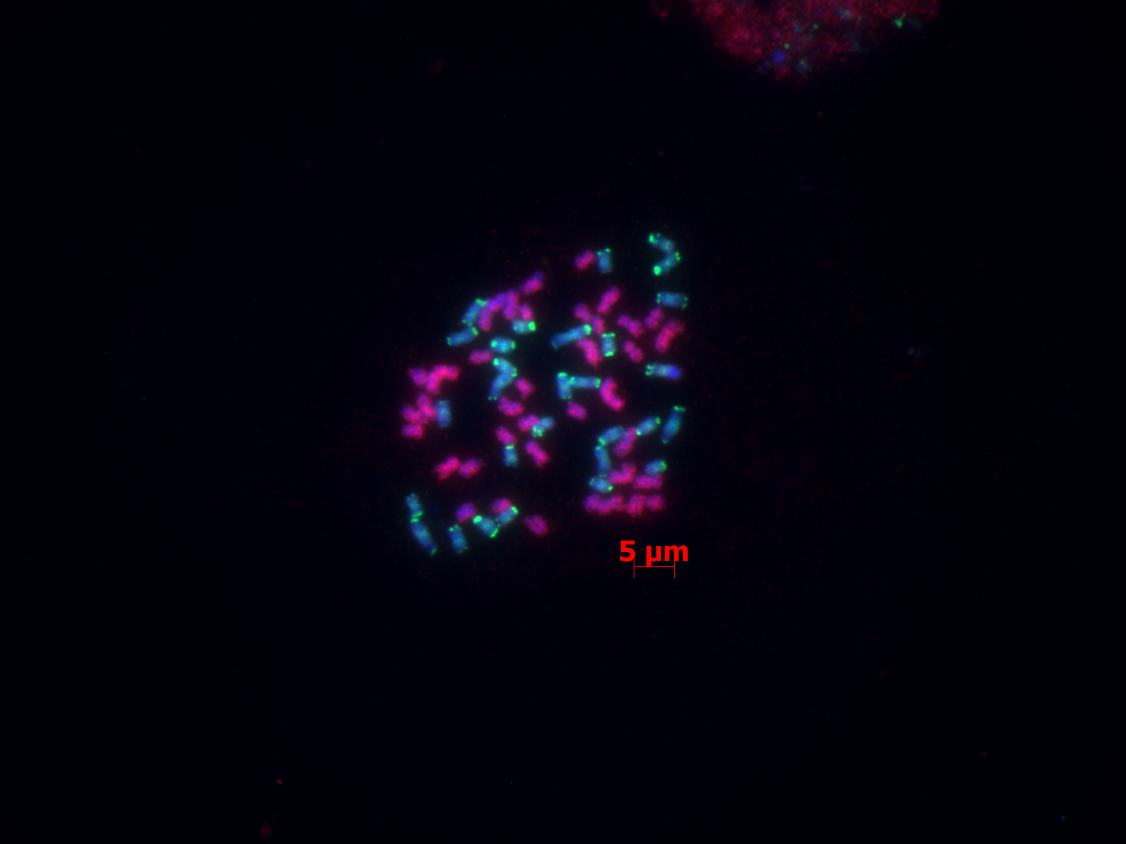


**YCE 96-45(F_1_)**: 29 chromosomes from *E*. *arundinaceus* and 40 chromosomes from *Saccharum* spp.

**Figure S5**


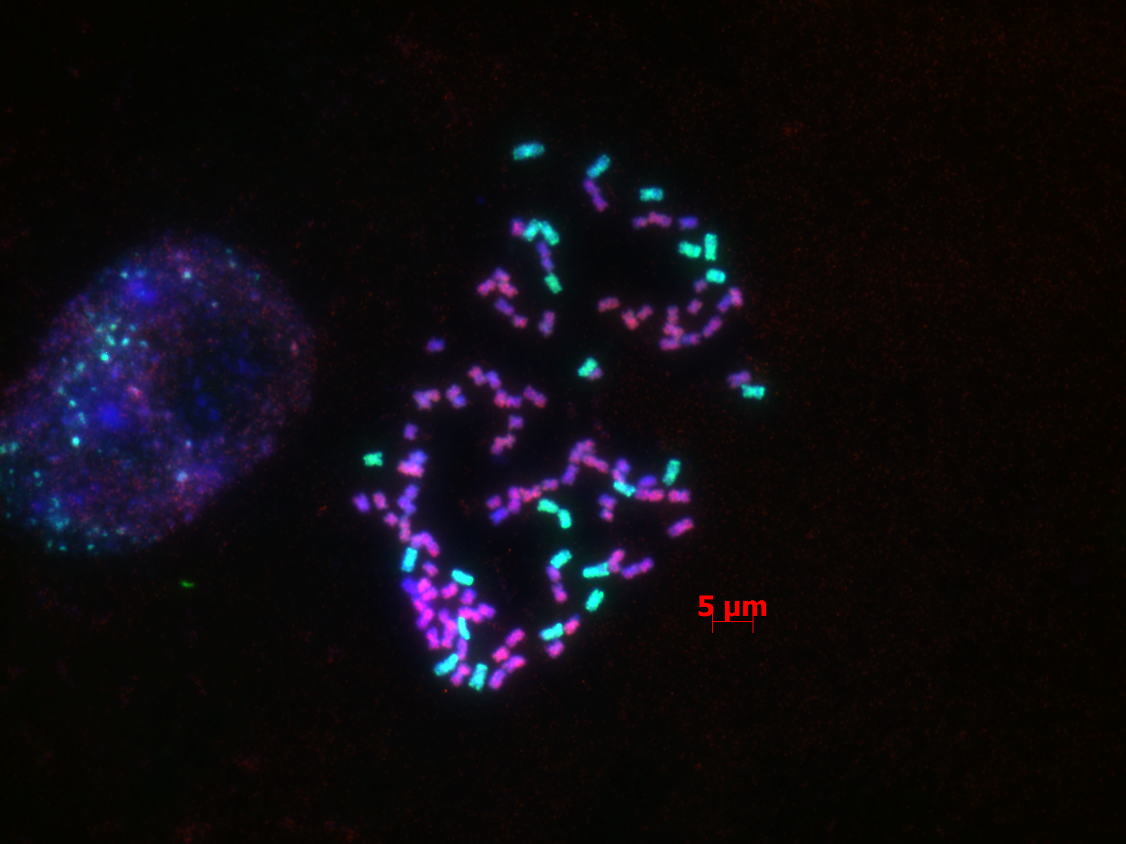


**YCE 01-33 (BC_1_)**: 27 chromosomes from *E*. *arundinaceus* and 93 chromosomes from *Saccharum* spp.

**Figure S6**


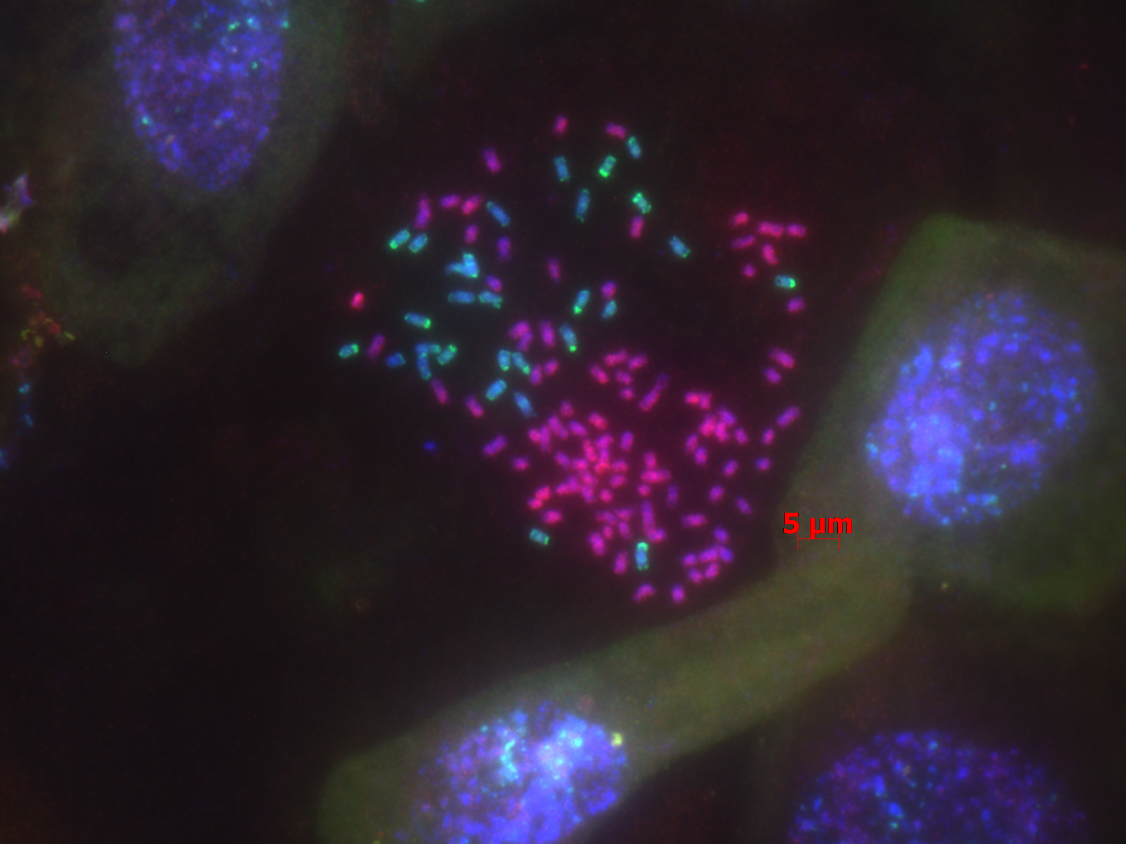


**YCE 01-46 (BC_1_)**: 29 chromosomes from *E*. *arundinaceus* and 96 chromosomes from *Saccharum* spp.

**Figure S7**


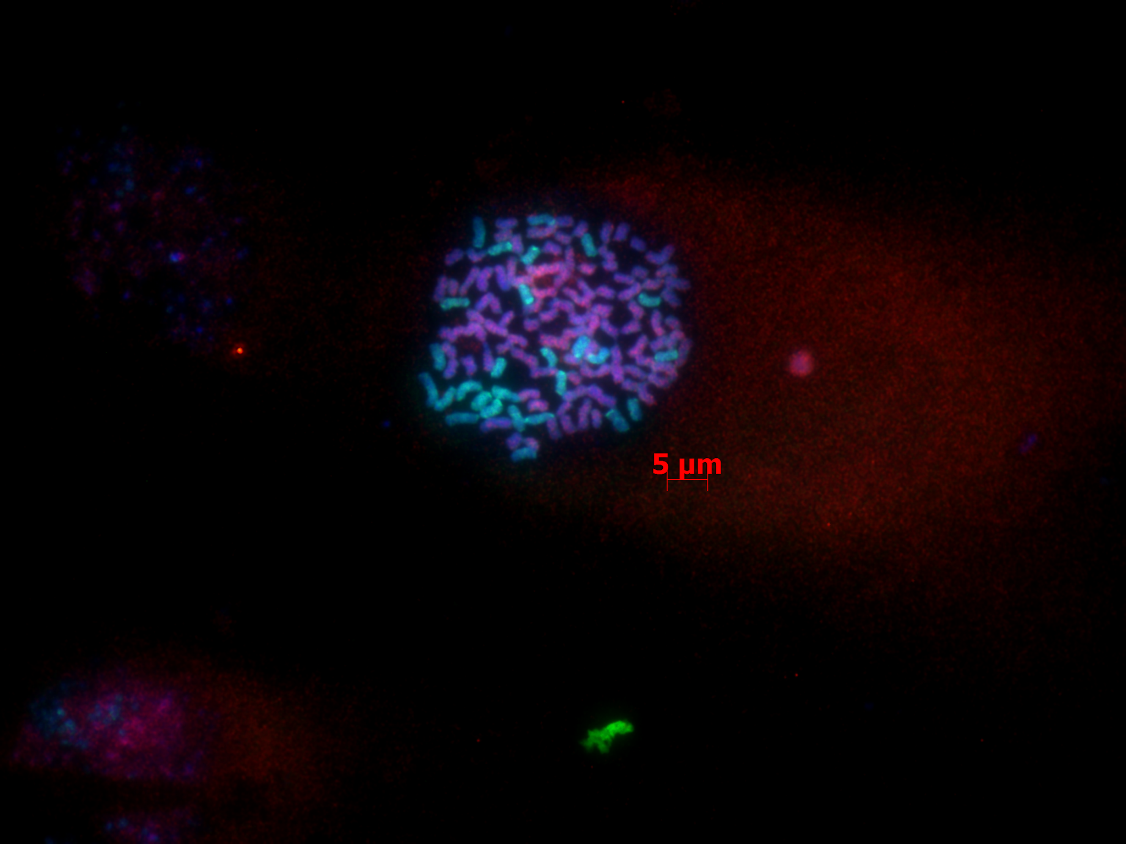


**YCE 01-48 (BC_1_)**: 27 chromosomes from *E*. *arundinaceus* and 93 chromosomes from *Saccharum* spp.

**Figure S8**


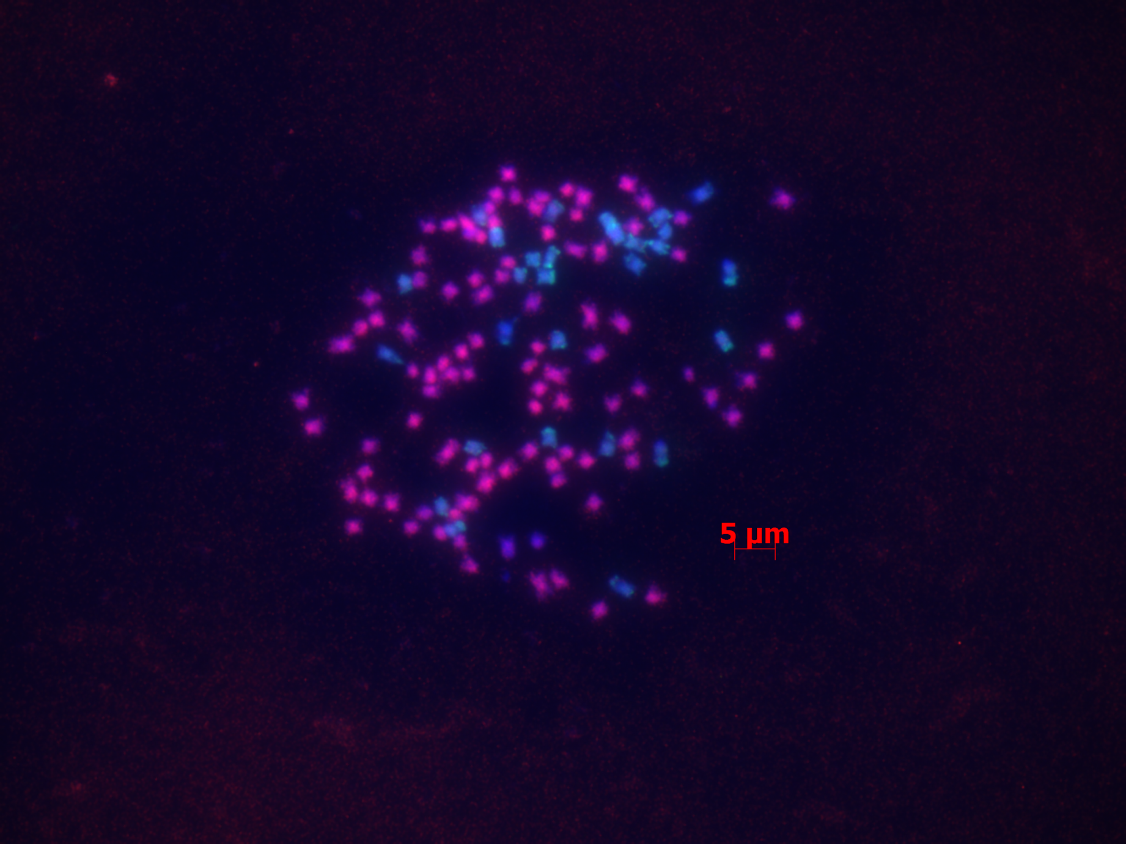


**YCE 01-63 (BC_1_)**: 28 chromosomes from *E*. *arundinaceus* and 97 chromosomes from *Saccharum* spp.

**Figure S9**


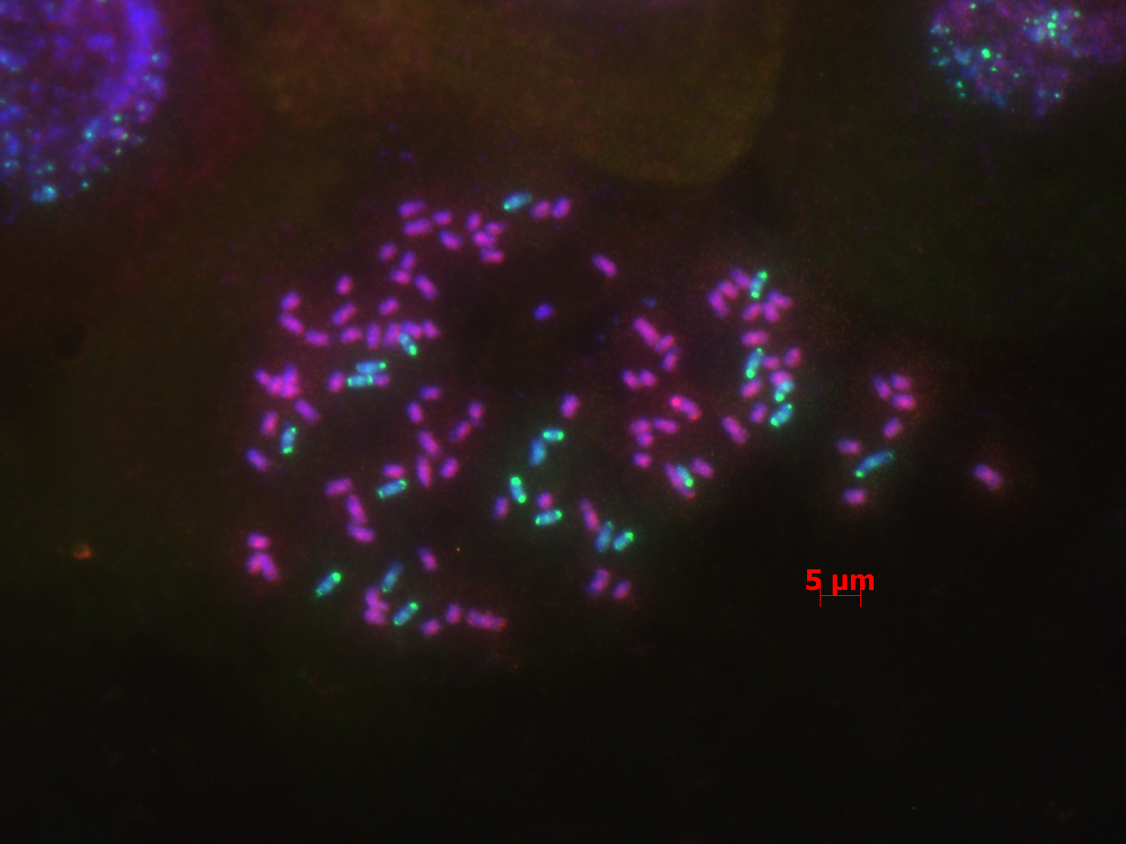


**YCE 01-99 (BC_1_)**: 23 chromosomes from *E*. *arundinaceus* and 95 chromosomes from *Saccharum* spp.

**Figure S10**


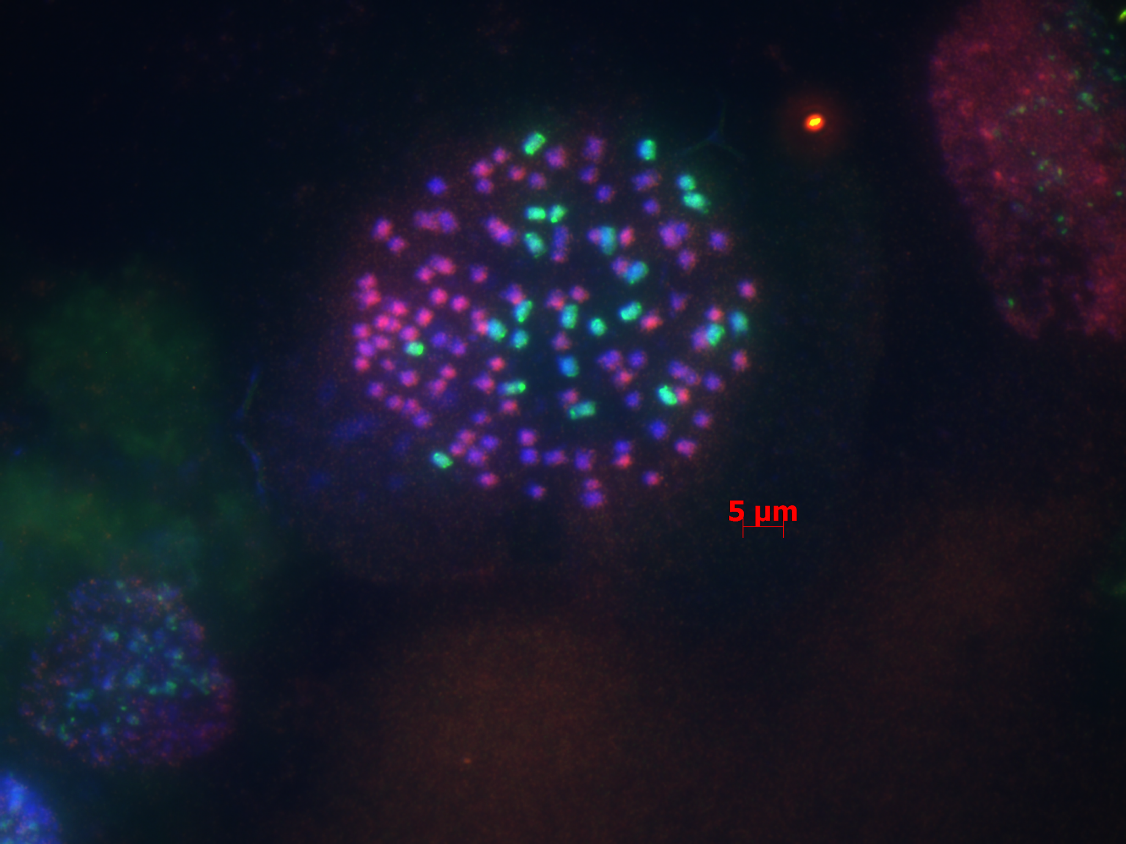


**YCE 01-105 (BC_1_)**: 23 chromosomes from *E*. *arundinaceus* and 94 chromosomes from *Saccharum* spp.

**Figure S11**


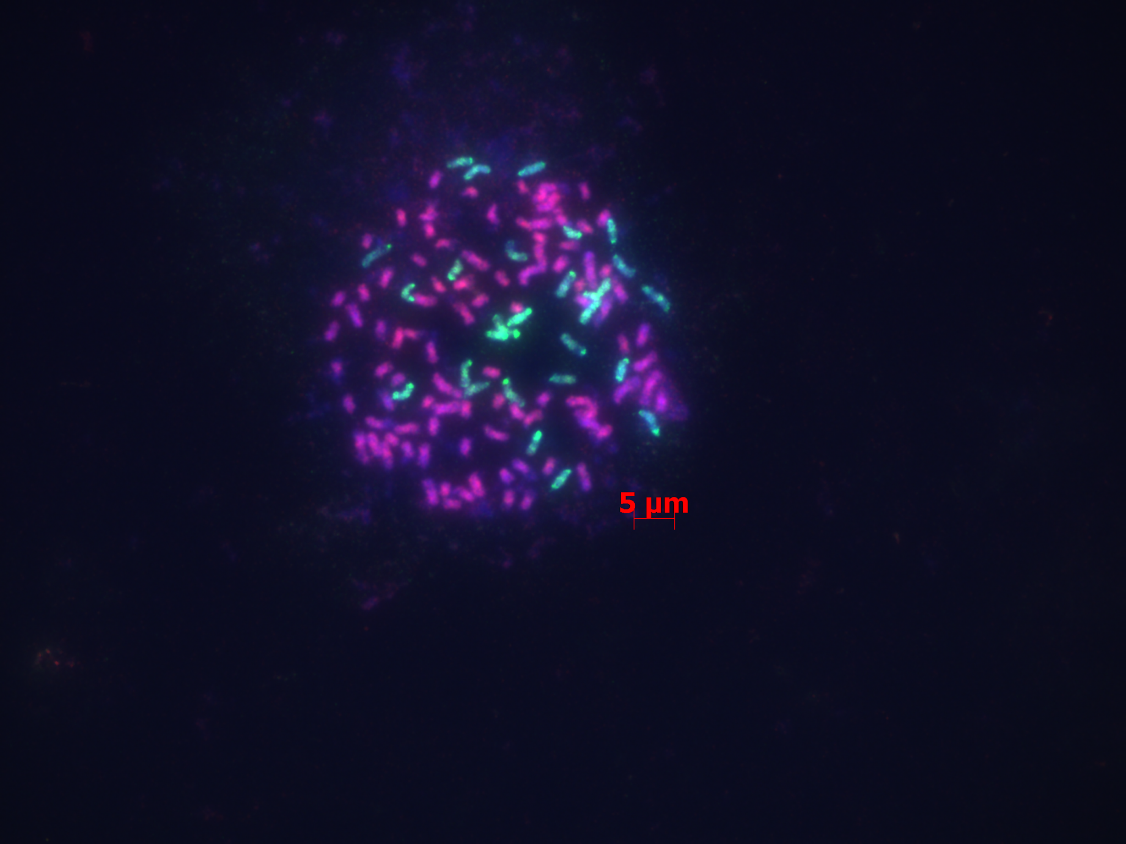


**YCE 01-116 (BC_1_)**: 28 chromosomes from *E*. *arundinaceus* and 94 chromosomes from *Saccharum* spp.

**Figure S12**


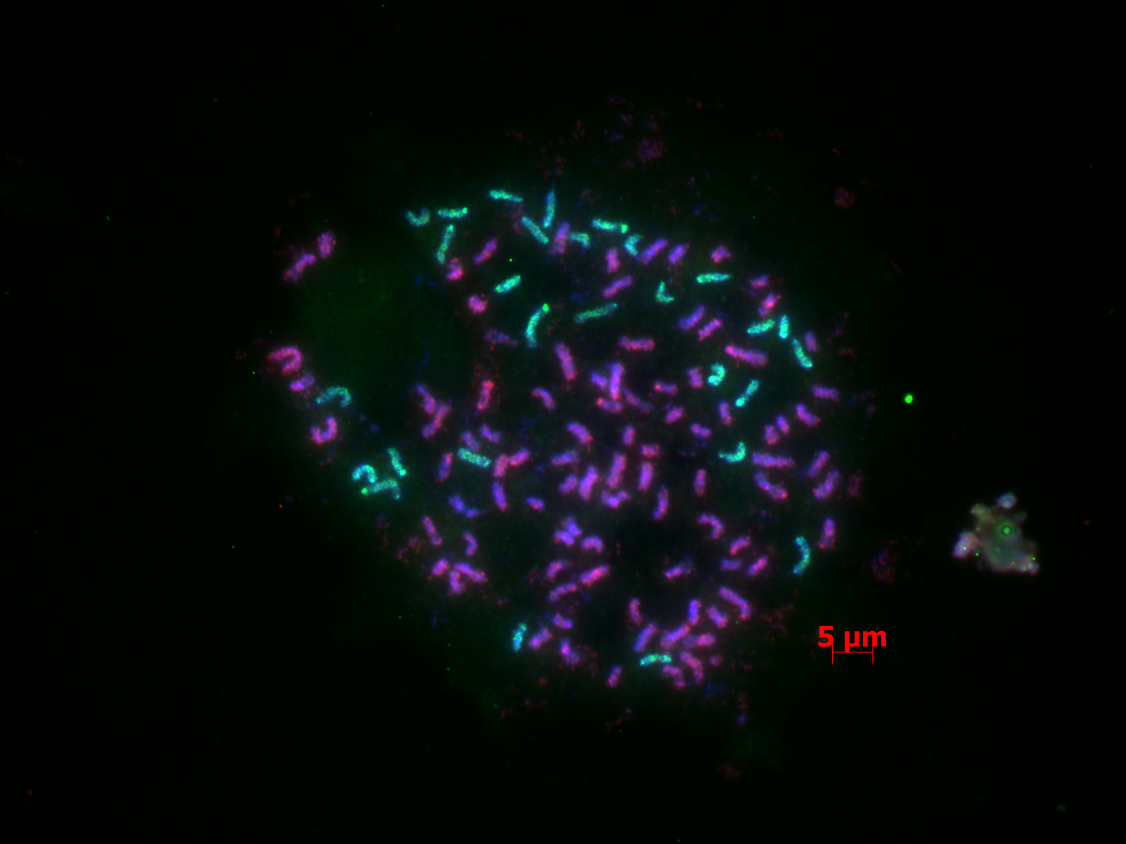


**YCE 01-134 (BC_1_)**: 28 chromosomes from *E*. *arundinaceus* and 93 chromosomes from *Saccharum* spp.
